# From a clinical case to a general methodology to analyze prosthetic joint failure, by micro- and nano-characterization with SEM of intra-tissue wear debris

**DOI:** 10.1186/s42649-025-00118-2

**Published:** 2025-12-15

**Authors:** Stefania Tarter, Lorena Maines, Giandomenico Nollo, Marco Molinari, Stefano Gialanella

**Affiliations:** 1 Department Industrial Engineering, University of Trento, Trento, 38123 Italy; 2 BIOtech Center for Biomedical Technologies, Department of Industrial Engineering, University of Trento, Trento, 38123 Italy; 3 Division of Orthopedics and Traumatology at Fiemme Hospital, A.P.S.S., Trento, Italy

**Keywords:** Debris, Wear, Methodology, SEM, EDX Spectroscopy, Hip prosthesis

## Abstract

Release of prosthesis debris at the tissue-implant interface is a major cause of aseptic loosening, a phenomenon that requires premature replacement of the prosthesis. The main objective of this paper is to propose a step-structured *modus operandi* for a reliable scanning electron microscopy (SEM) analysis of debris released from prostheses into the surrounding tissues. Following a proven methodology for the analysis of this wear debris would allow research and hospital laboratories to reduce time and obtain results associated with a common protocol and consequently, more comparable results. For developing the methodology, we chose the clinical case of a hip prosthetic revision, in which a Cr-Co head misalignment caused the wearing out of the polyethylene acetabular insert and a partial wear of the Ti-6Al-4 V acetabular cup. Samples of periprosthetic tissues, after being partially digested in a KOH basic solution, were investigated in vivo and in situ with SEM observations and Energy Dispersive X-rays Spectroscopy (EDXS) analyses. Although developed from a specific case study, this methodology is compatible and applicable to other standard cases as well. Regarding the set of samples we selected, a complete set of micro- and nano-structural analysis, compositional spectra and high-resolution images have been acquired, showing the morphology of the debris involved, and the agglomeration phenomena occurring in the tissue. The proposed protocol complements previous studies on tribological phenomena, underlying debris production at the tissue-prosthesis interface, best digestion techniques for fragment isolation, and nanotoxicology.

## Introduction

Biophysical phenomena that occur at the interface between the prosthetic components and the surrounding tissues strongly determine the difference between successful and unsuccessful arthroplasty surgery. The causes of the generation of such micro-nanometric fragments are partial or absent osseointegration of the prosthesis, tribo-mechanical factors, such as micro-displacement or misalignment of the components, but also the simple aging process of the prosthetic surface associated with corrosion and wear phenomena between articulating surfaces, non-articulating surfaces or between modular interfaces of the joint arthroplasty (Jones and Buckle 2020; Keegan 2007). The unwanted presence of these fragments leads to aseptic osteolysis and consequently to aseptic loosening, which is the primary cause of prosthetic revision. In (Sadoghi et al. 2013) Sadoghi et al. reports that 55.2 percent of the total number of revision surgeries conducted within one year after total hip arthroplasty are caused by aseptic loosening, 11.8 percent by dislocation or instability, and the remaining by causes including septic loosening, implant wear, periprosthetic fracture, implant breakage and other technical problems. Regarding the loosening process, a review can be found in Revell (2008), that delves into the role of such wear particles, associated with the subsequent presence of lymphocytes and macrophages.

It is worth introducing a brief overview of the types of materials used in prosthetic components, as this is closely related to the amount and type of debris involved.

As for polymeric materials, Ultra-high-molecular-weight polyethylene (UHMWPE) is one of the most common, because of its self-lubricating ability and elasticity. Its main use in biomedical engineering is to produce bearings in total joint arthroplasty, and in general, components in the articulating areas of the prostheses, as acetabular cups of hip prostheses, tibial plates, and patellar surface of knee prostheses. It consists of polymerized monomers, forming long chains, organized to some extent in crystalline domains. This results in redox reactions involving body tissues, increases elasticity and decreases the coefficient of friction. Polyethylene itself is an inert material toward cells, but the fragments, which can reach larger sizes than metal fragments, may induce foreign-body reactions: this is so since it is a material resistant to enzymatic attack and therefore the fragments cannot be degraded or reduced to simpler units. This triggers enzymatic production that is capable of self-feeding and amplifying over time, inducing inflammation and necrosis of surrounding tissues (Katz et al. 2004; Hodges et al. 2021). To reduce the particulate release from UHMWPE, several routes have been followed, ranging from totally replacing the material with metallic or ceramic bearings, which however still release other debris and have the disadvantage of being less versatile systems; to making the polymer more wear resistant, through various techniques, the most promising of which is cross-linking. Laurent et al. (2008) demonstrated how Highly Cross-linked Ultrahigh Molecular Weight Polyethylene (HXLPE) has significantly better wear properties than conventional UHMWPE. In addition to showing a better resistance to oxidation, due to fewer free radicals, this material generates significantly less debris, with a lower size. After a hip simulator test, SEM analysis demonstrated that HXLPE produced 97% fewer particle overall and 94% fewer particles in the smallest particle range detected (0.10–0.15 µm) with respect to the conventional UHMWPE. In Tsukamoto, et al., (2017), the impact of HXLPE on wear-related reoperation rate was investigated, focusing on a material produced by a single manufacturer and comparing it to conventional polyethylene (CPE). The study retrospectively examined the 12 year-clinical history of a group of patients, with cementless total hip arthroplasty with Cr-Co head. It resulted that, thanks to the significantly reduced wear rate of cross-linked polyethylene (0.035 mm/y versus 0.118 mm/y of the common one), the group with bearings components in HXLPE showed a cumulative survival rate at the 12-year follow-up of 100%, with respect to 91.4% of the CPE group. As far as the osteolysis incidence is concerned, the cumulative survival rate at the 12-year follow-up was 100% for the HXLPE group and 36.2% for the CPE group.

Polymethylmethacrylate, PMMA, is obtained by free-radical polymerization of the monomer, methyl methacrylate, in a solution and also containing microgranules of barium sulfate. During polymerization, the cement, inserted into the anchorage zone of the prosthesis, takes on a pasty consistency. Once this process is complete, it solidifies and locks the prosthesis in place. One of the main problems associated with this material is that PMMA polymerization occurs exothermically. This results in thick and large cement layers, in the formation of pores, detrimental to mechanical properties, damage to blood vessels present and occasionally thermal necrosis of bone. This may determine the disruption of the bone-cement interface, leading to micro-movement, wear, and aseptic loosening. Another problem associated with this material is the tendency to develop bacterial contamination. A possible remedy to this latter inconvenience is loading PMMA-based bone cements with antibiotics (antibiotic-loaded bone cements, ALBCs). However, the introduction of medicals could be incompatible with the therapy administered to the patient, and in general, this approach may lead to or promote antibiotic resistance. For this reason, antibiotic-free PMMA-based cements with antibacterial properties have been developed, the types developed in recent years are varied and very promising, Bistolfi et al. in (2019) set out a review of them, describing cements loaded with nanoparticles (made of silver or graphene oxide), or organic antibacterial agents. They contain the combination of an inorganic, antibacterial phase with a bioactive one, limiting bacterial proliferation and improving adhesion to the bone. The latest generation are PMMA bone cements nanocomposites proposed in Phakatkar et al., (2020), containing 2D magnesium phosphate (MgP) nanosheets and hydroxyapatite (HA) nanofibers. These nanocomposites, used in combination, improve the mechanical properties, bioactivity and cytocompatibility of PMMA, proving to be the candidate filler for the next generation of cements.

Another very interesting category of materials is that of ceramics, like aluminum oxide (alumina-Al_2_O_3_), and zirconium oxide (zirconia—ZrO₂). On the combination of Al_2_O_3_ with ZrO₂ is based the latest generation of material systems, known as zirconia-toughened alumina (ZTA), in which alumina is the primary or continuous phase (70–95%), toughened by zirconia, present in the composites with concentrations ranging from 5% up to 30% (Kluess et al. 2014). Ceramics are biocompatible and nontoxic materials and are used to overcome the major issue of polyethylene wear, because of their higher hardness, strength and heat and, therefore, wear resistance. These properties, however, are associated with almost zero ductility and high brittleness, which lead to the possibility of fragmentation and makes machining complicated. Ceramic materials can be used as bulk materials or as coatings. This can be a successful solution for patients with metal allergies or a way to reduce or inhibit the release by metal ions (Kurtz et al. 2014; Gibon 2016).

In this study, we will focus on metallic debris, easily visible using SEM images thanks to the high atomic number of its constituent elements. The most common metal alloys in this field are stainless steels, titanium alloys and cobalt-chromium alloys (CrCo and CrCoMo). These alloys are usually highly resistant to corrosion, as they form a self-passivation layer on their free surfaces. Therefore, they are more prone to mechanical wear than electrochemical corrosion. In particular, the Ti-6Al-4 V alloy proves particularly resistant to corrosion as it forms a TiO_2_ layer on its surface. Due to the toxicity of vanadium, attempts have been made to replace it, in such a widely used alloy, with iron (Fe) and niobium (Nb), thus trying to increase mechanical strength as well as biocompatibility. The resulting alloys, Ti-5Al-2.5Fe and Ti-6Al-7Nb, allow a better implant/bone stress distribution because of the greater dynamic hardness and the lower elastic module. In particular, Fellah et al. concluded in (Fella et al. 2014) that the alloy Ti-6Al-7Nb shows similar friction and wear performance as Ti-6Al-4 V, notwithstanding its different structure and composition. Molybdenum concentrations in excess of 10% are typically faced in β-Ti alloys. They have β-Ti polymorph stable at room temperature, featuring a significantly lower Young’s modulus (∼70 GPa versus ∼110 GPa of α + β titanium alloys such as Ti-6Al-4 V) and behavior closer to real bones. Moreover, they are easier to shape but no longer used because they produce a high number of debris (Merola and Affatato 2019; Yang and Hutchinson 2016).

Chromium-cobalt alloys have an excellent resistance to corrosion and wear and produce less debris than steel titanium alloys. In fact, the linear wear rate of CoCr alloy is about 0.1 micron of material per year (corresponding to 10^6^ cycles of the tribometer’s motion), whereas the wear rate of 316L stainless steel and Ti-6Al-4 V are in the order of 0.2 microns and 1 micron per year (10^6^ cycles), respectively. Because of reduced linear wear production, CoCr alloys have often been used in metal-on-metal (MoM) articulations, replacing metal-on-polyethylene (MoP) ones (Buford and Goswami 2004; Davidson 1993). However, it is essential to remember that CoCr particles are toxic at high concentrations: in patients with metal-on-metal hip implant, there is a positive linear correlation between elevated levels of circulating Co and Cr and lymphocytic reactivity (Hallab et al. 2005; Jacobs et al. 2003).

More in general, mechanical wear that occurs between metallic components produces debris whose size varies from tens of nanometers to a few micrometers. In the former case, the debris rarely produces inflammatory reactions and macrophage driven osteolysis, they are also so small to be easily excreted, transported, and taken up systemically. These metal particles can also corrode and therefore disappear. However, they do still produce biological response, which can result in a pseudo-tumoral formation, osteolysis and aseptic loosening of the prosthesis as well as necrosis of surrounding soft tissues.

The research carried out so far has achieved excellent results in microbiological, histological, and microscopic analysis. Nine et al. (2014) propose a thorough review, on studies prior to 2013, concerning the microscopic characterization, tribological analysis, and biological response of wear debris from knee and hip replacements. The principal steps of particulate isolation exposed are sample delipidation and tissue digestion (Slouf et al. 2007), dilution, centrifugation and protein separation (Catelas et al. 2001), ultrasonication and particle separation (Lapcikova et al. 2009) applied on both periprosthetic tissues (in vivo) and simulated body fluid (in vitro). Whether debris generated within a human body or reproduced in the laboratory following tribological studies, these studies focus on particulate matter in general completely isolated from tissue and the most recent ones show follow this trend too (Schappo et al. 2018; Crainic et al. 2019; Crainic, et al., 2020). Depending on the materials involved, various digestion approaches like acidic, alkaline, enzymatic (Visentin et al. 2004; Tipper et al. 2006) have been proposed and studied considering a trade-off between good isolation and reduced particulate damage. The choice to study particulate samples fully digested and isolated from surrounding tissues is justified by the fact that it allows for more in-depth characterization and in general allows for better details of 2D and 3D images.

Because of that, most articles whose purpose is to analyze debris from prosthetic components attempt to isolate the particulate completely. The literature presents several articles (Espallargas et al. 2017) on the in-situ generation of reaction films and tribo-metallurgical transformations on MoM hip joints, responsible for debris production. There are, nevertheless, very few studies that, as in our case, choose to study such debris in-situ as well (Topolovec et al. 2013), thus gaining the considerable advantage of less damage to the particulate surface and assessing the possible agglomeration effects occurring within the tissue.

In Authors’ view, the need for a general protocol to ensure a standardization of wear debris characterization is mandatory.

The aim of this research is to fill this gap by proposing a stepwise methodology that allows the study of wear fragments still partially embedded in the tissue in which they were released. A study of intra-tissue particulate matter, conducted in the light of a controlled procedure, obviously allows a less complete characterization of individual fragments, but still affords the possibility of evaluating aspects related to the way biological tissues respond to their presence. More importantly, it allows control of the digestion process, if chosen, during its implementation and in selecting the best digestive agents’ concentrations, an aspect that is particularly important since inappropriate isolation method may have detrimental effects on the size, shape, and number of particles, as it could alter their morphology or cause them to agglomerate. This study proposes a general protocol, starting with the study of metal debris generated by a hip replacement, and aims to pave the way for the implementation of other methodologies suitable for each of the other materials involved in arthroplasty.

This paper presents a detailed explanation of the methodologies developed, and the experimental parameters. We propose a flow chart summarizing its main points, which is easily adoptable to similar samples, in order to optimize the analysis procedure and to reduce the test time, a prerequisite for managing massive volume of samples, as is often the case in hospital contexts. The critical discussion of the results will contribute to clarifying the applicability fields.

## Methods

### Materials

The specimens analyzed came from biopsies taken during hip, shoulder and knee prosthetic revision surgeries. The collection of these biopsies took place in the context of the RIFEndO research project, which involved three Italian hospitals in the Autonomous Province of Trento (Cavalese, Rovereto, and Trento, Italy), with the aim of studying the role of the tissue-prosthesis interface in bone endoprosthesis failure. These samples have been studied in previous works, (Valentini 2008; Miori 2008), which inspired the present research.

The work by Valentini (2008) focused on identifying experimental methods to determine the causes responsible for arthro-prosthetic failure. Microbiological, histological, and ultrastructural analyses were performed on intra-operative samples (biopsy samples of peri-implant tissues, samples of prosthesis-bone interface material, joint fluid, and components of arthro-prostheses) and on the explanted prostheses themselves. Regarding the use of scanning electron microscopy in association with energy dispersive X-ray spectroscopy (SEM-EDXS), microstructural analyses were conducted on prosthesis surface, sections and periprosthetic tissues. Compositional characterization of periprosthetic tissue sections allowed confirmation of the presence of particulate matter coming from the wear mechanisms and processes typical of these implants. Optical microscopy was used to conduct preliminary observations at medium magnifications, on histological sections hematoxylin–eosin stained (H&E). Observation of the tissue section by electron microscopy and EDX spectroscopy, carried out at medium–low magnifications mainly, have been important for detecting the presence of polyethylene, cementum and metal fragments.

Miori's study (Miori 2008) focused on optimizing procedures for characterizing prosthetic wear fragments found in peri-implant tissues, starting from a literature survey on previous works on the subject. It mainly compared and tested methods for isolating polymer and metal debris and therefore facilitated their analysis with optical and scanning electron microscopy techniques.

As far as concerns our research, its main purpose is to devise a general stepwise methodology, applicable to the wider possible range of specimens and suitable, in particular, for analyzing prosthetic joint failure with SEM, by micro- and nano-characterization of intra-tissue wear debris.

We validate the developed approach using a real life specimen, mainly consisting of foreign body granulomas extracted from the joint pseudo-capsule, which showed a large amount of metal and polyethylene debris. The origin of this sample is the clinical case RO 008, related to an uncemented left hip prosthesis, previously observed, in combination with other clinical cases, by Miori and Valentini (Valentini 2008); Miori 2008). The failure of this clinical case was due to the decentralization of the Cr-Co femoral head, compared with the Ti-6Al-4 V cup with polyethylene cotyloid insert. This decentering, diagnosed in the hospital by pre-explant radiography, was responsible for wear of the prosthesis components and production of debris resulting in osteolysis and aseptic loosening, after which the patient underwent prosthesis revision, 17 years following the first implantation.

### Experimental design and instruments

The SEM used for the analyses is a JEOL—JSM—IT300LV instrument. We operated it in high vacuum mode at the voltage of 20 kV, with a working distance of about 10 mm.

The SEM instrument is equipped with two electron detectors: Secondary Electron Detector (SED) recommended for fine structure and topographic details (SE images); the Backscattered Electron Detector (BED) for images that give information on sample composition (BSE images). This SEM is also equipped with an EDXS C/U detector, using software for data acquisition, and for analyzing the spectra both qualitatively and quantitatively. X-ray elemental maps were also acquired, to assess the spatial distribution of the detected elements.

For the EDX spectral analysis, the Max Throughput, which is the threshold of kilo-counts per second (kcps), was set to 60 and the typical working values were 20 kcps. The working time was 2 min in Manual Mode.

For EDX mapping, the Max Throughput was set at 275 kcps and the typical working value ranged from 50 to 90 kcps. The working time was 2 min in Manual Mode.

### Methodology

One of the main objectives of this research is to develop a general methodology, applicable to a wide range of samples with debris, and featuring hierarchical steps that can simplify the work and optimize the time for searching wear debris in biological samples. This approach is particularly useful in cases where the matter size and type do not allow for its immediate identification within the tissue.

**Fig. 1:**
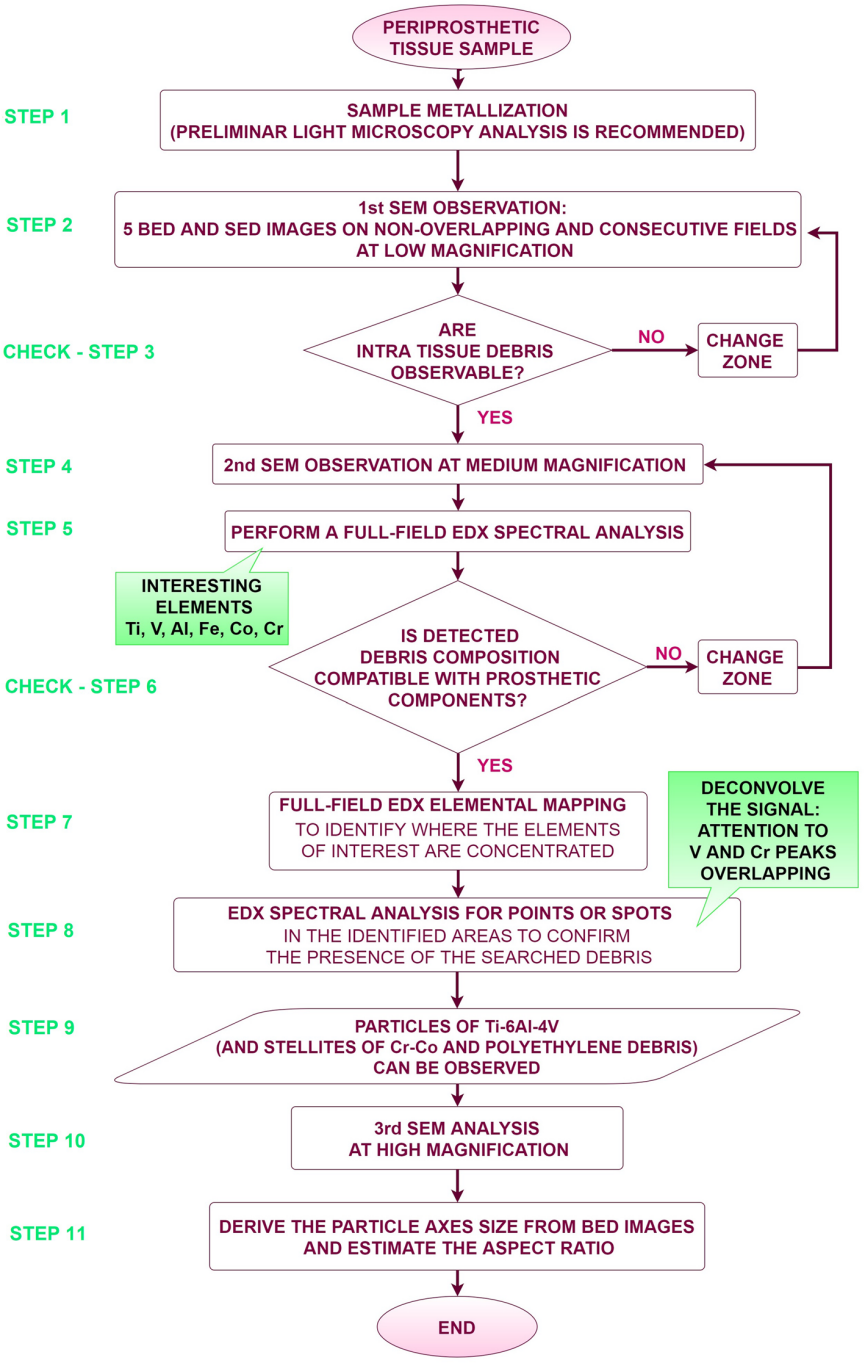
Flow chart of the proposed modus operandi

This streamlined protocol is intended to be a guide to those who need the support of a rigorous, detailed, and tested *modus operandi* in both research and hospital diagnostic and testing laboratories. The main steps of the proposed methodology will be described in detail below (see Fig. 1, for the relevant flow chart).

#### Sample preparation

Sample preparation was described in detail in Valentini (2008). We will herewith report on the main steps for ease of reading. The biopsy samples, embedded in kerosene, were microtome sectioned into slices of about 2 μm thickness and placed on the side of the filter that, upon microscopic observation, appeared smoother and more regular. Kerosene removal was obtained by double treatment with two successive applications of xylol in glass petri dishes for 5 min. The xylol-treated samples were dried first with blotting paper and then in air, to dry up completely in a fume hood. At this stage, samples were covered with glass Petri dishes, to prevent the deposition onto the filter of external particulate matter. Among the various samples related to the clinical case RO 008 and specifically prepared for electron microscopy, those digested for 1 h were chosen, with KOH at a minimum concentration of 0.2 M and a maximum concentration of 2 M. This concentration and the one-hour digestion time were not exceeded since, as pointed out by Catelas (2001), at higher concentrations this reagent changes the size of the metal particles, completely jeopardizing the morphological and quantitative analysis. All digestion procedures were carried out at room temperature in a chemical fume hood. Tissues prepared with KOH showed partially digested portions and others still intact. The fact that this digestion technique did not allow complete isolation of particulate matter gave this research the opportunity to observe particulate agglomeration phenomena more closely, so that we could determine the size and morphology not only of the debris emerging well from the tissue, but also of the debris partially or completely embedded into layers of organic material.

Eosin and hematoxylin staining and optical light microscopy analysis are necessary to localize in the sample only those sections with significant particulate matter, which SEM analysis will characterize at higher resolution.

Before starting with SEM characterization, the sample must be metal coated, to prevent electrical charging. Surface coating would also protect the organic sample, made conductive, from overheating and degradation by the electron beam bombardment. The specimens were anchored with a double-sided carbon conductive adhesive tape onto electron microscopy specimen holders (stubs) and then gold coated with an Emitech K500x sputter coater.

#### 1st SEM observation

For the first SEM observation at low magnification, at least 5 consecutive, non-overlapping zones on the sample should be identified, of which five low-magnification secondary electron (SE) and backscattered electron (BSE) images should be acquired to determine where to narrow the field of view for subsequent analyses.

In fact, SE images are useful for getting a better overview of the sample at low magnification. However, it is the BSE images that show us which areas are of real interest based on the concentration of metal particles present.

#### Check step

Debris that is metallic or consists of elements with higher atomic number (Z) than the organic tissue matrix appears in BSE images as spots with a brighter contrast on a dark gray background. If such fragments are observable, then it possible to proceed with second observation. Otherwise, it is necessary to locate another area of the sample (back to step 2).

#### 2nd SEM observation

At this stage, the sample can be observed at a medium magnification level. By means of the BSE image, it is possible to choose an area on the sample that is rich in particulate matter.

#### Full field EDX spectral analysis

A full-field EDX spectral analysis should be conducted in this area. The purpose is to identify the elements present in the sample. Regarding debris from prosthetic materials, the metal elements of interest are mainly: Ti, V, Al, Fe, Co, Cr. The element used for metallization (usually Au) and carbon C generally are removed from the list of elements to be quantified, because it is not easy to evaluate the contribution of the coating, the organic matrix and the embedding medium, whose pervasive presence would make the percentages of the elements of interest negligible.

#### Check step

If the elemental composition of the debris is compatible with the source prosthetic components, then the chosen zone is appropriate. Otherwise, a change in zone is recommended, proceeding again with a general observation at medium magnification (back to step 4.)

#### Full field EDX elemental mapping

To understand where the elements of interest are located within the portion of the sample under observation, we proceed with a full-field EDX elemental mapping. Based on similar former experience, we chose to pay attention to normalized elemental concentrations higher than wt.1%, because lower ones may not indicate the presence of the relevant element in the sample, but rather a surface contamination.

#### EDX spectral analysis for points or spots

EDX spectral analyses have been conducted either in full-field mode or on individual points or spots of the selected regions.

#### Presence of debris

We have at this stage the necessary information, regarding microanalysis and localization of debris within the sample, to proceed with further SEM observations of the wear fragments at higher magnification, in order to investigate in greater detail their morphological features.

#### 3rd SEM observation

The latter observation at high magnifications allows, using both SE and BSE images, to observe the particles closely and to assess their morphology, surface topography, and size. It is important, at this stage, the assessment of the quality of the digestion process, if present, by observation of how much detritus emerges from the organic matrix. In case the sample was only partially digested, and the organic matrix was only superficially modified, an important aspect that can be noted is the presence of agglomerates.

#### Morphological information collection

Once well emergent particles from the biological tissue matrix are identified on the BSE images, major and minor axis measurements can be made to estimate the aspect ratio.

Under conditions where the tissue has been fully or just partially digested and the particles are clearly visible and individually distinguishable, automated image analysis can be performed to calculate dimensional and morphological parameters such as equivalent circle diameter, area, perimeter, aspect ratio, roundness, and form factor.

## Results

The results obtained according to the proposed methodology are described in the next session.

### 1st SEM observations at low magnification

During the first SEM observation, it was possible to figure out which of the samples prepared in Phakatkara et al. (2020) and Kluess et al. (2014), using the techniques reported in Kurtz et al. (2014), are suitable for in depth analysis.

Samples digested with nitric acid (HNO_3_) have a rather irregular and discontinuous structure: they show alternating portions of intact tissue with portions where the digestion process is so advanced that it has removed the tissue itself, creating fractures. This category was therefore excluded, preferring a type of sample in which the digestion process was less aggressive on the tissue.

**Fig. 2:**
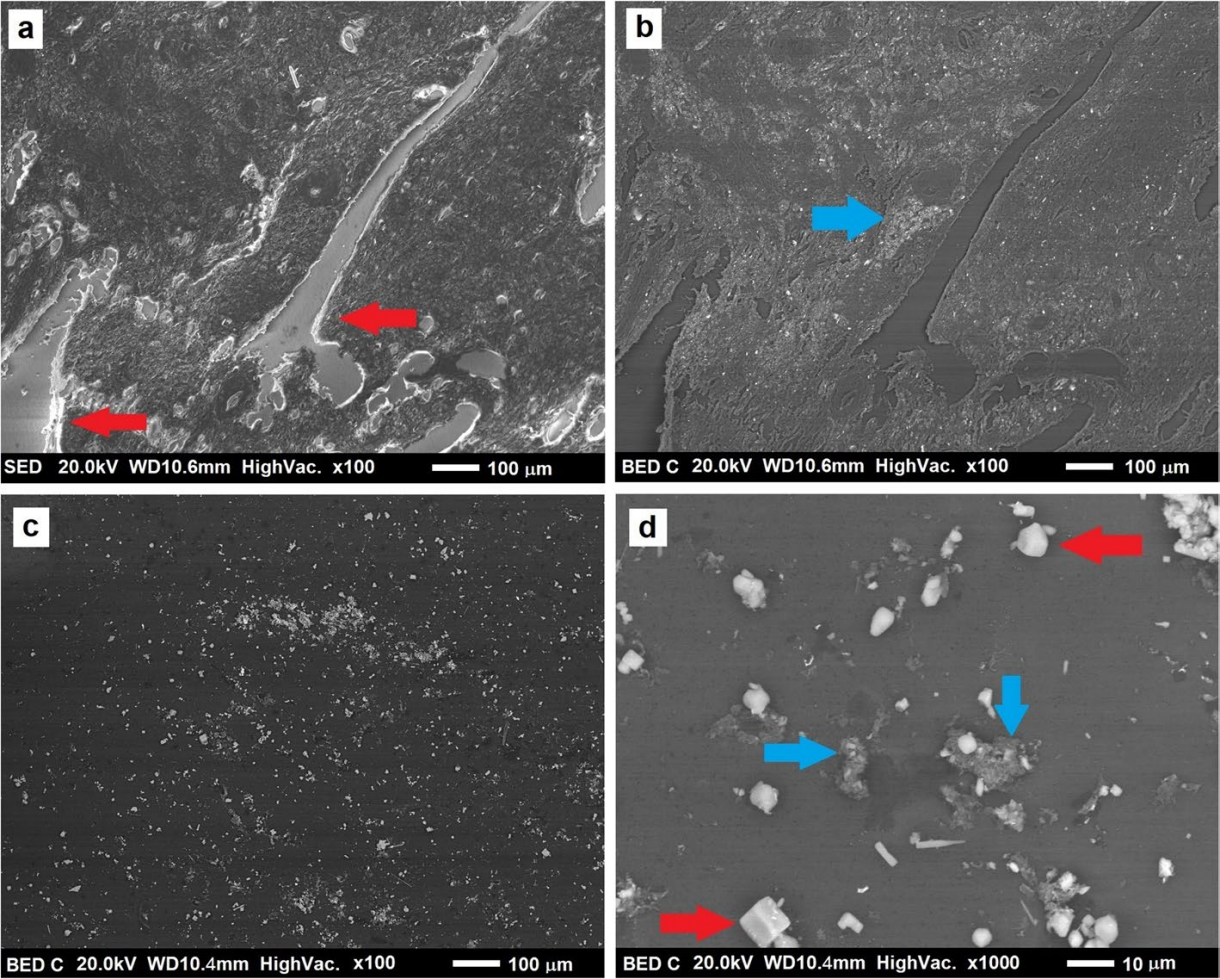
**a** Shows an SE image of an HNO3 digested sample at low magnification. The brighter contrast of the boundaries of cracks and holes is due to charging effect (red arrows). BSE image of an HNO_3_ digested sample at low magnification: metallic particles are well visible on the grey background of the tissue (see the blue arrow, **b**. BSE image of a NaClO digested sample at low magnification (**c**). A medium magnification BSE image, with details of the NaCl residues (cubes pointed by red arrows). Tissue residues are pointed by blue arrows. The other irregular, white particles are metal debris (**d**)

SE images generally show irregularity on the surface, especially near the fractures. In Fig. 2a the fractures are indicated with red arrows. In Fig. 2b blue arrows point at the metal particles (brighter contrast on a grey background) detected in the sample.

Regarding the samples digested with sodium hypochlorite (NaClO), the metal particulate is mixed with salt residue (NaCl) recognizable by the typical cubic shape and left behind by the digestion process. At low magnifications, as seen in Fig. 2c, they are difficult to distinguish from metallic particles, as they also appear as white dots on a dark background. They are easier to spot in a medium magnification image, where the tissue is almost entirely digested, except for a few shreds of tissue closer to the clusters of particles (see Fig. 2d). This category was also excluded, because it did not give the opportunity to assess the interaction between tissue and debris.

**Fig. 3:**
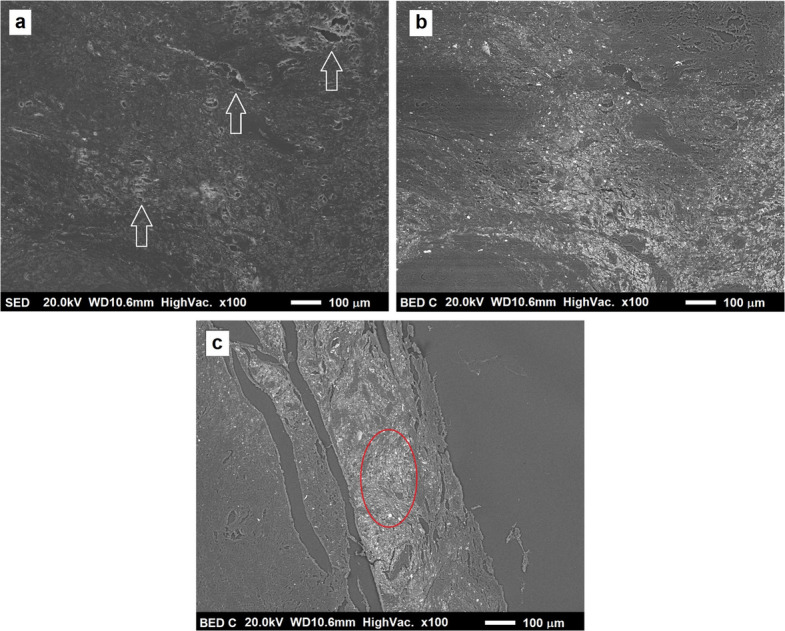
**a** Shows an SE image of the central part of a KOH digested sample at low magnification. White arrows indicate small fractures in the specimen, quite small if compared to HNO_3_ sample’s ones. BSE image of the central part of a KOH digested sample at low magnification (**b**). A BSE image (**c**) of the same sample as in (**b**), observed in an area near the edge. The area inside the circle in this part of the sample is particularly rich in debris and was chosen for further analysis

The last category of analyzed samples includes those digested with potassium hydroxide—KOH. The surface of such samples is sufficiently regular to acquire satisfactory BSE and SE images, without particularly pronounced charging effects that would compromise analysis at higher magnifications and currents. The periprosthetic tissue was digested by this process only partially, at the surface level, leaving particulate matter to emerge in some places, in others allowing its observation only by BSE images. The samples show continuity across most of the surface and only a few fractures along the edges (indicated by white arrows in Fig. 3a), caused by damage due to the digestion process, and less large than HNO_3_ sample’s ones. No difference was found between samples digested in aqueous solution of KOH with a 2 M concentration, compared to those digested with 0.2 M solution. These samples were chosen for subsequent analysis at higher magnifications.

### 2nd SEM observation at medium magnification and EDX analyses

The selected area was subjected to a second observation at higher magnifications. The BSE image (Fig. 5a) shows the presence of metallic particles, recognizable by their lighter contrast with respect to the background, indicating higher atomic number composition.

We confirmed by full field EDX spectral analysis that these particles are made of the same metallic elements present in the prosthesis. The results obtained, reported in Table 1 detailing the normalized mass values, show the presence of titanium, aluminum, and vanadium, originating in the acetabular cup in Ti-6Al-4 V, and trace amounts of cobalt originating from the Cr-Co femoral head.

**Table 1:** The full field EDX spectral analysis confirmed that the particles in Fig. 9 come from the prosthetic implant: acetabular cup made of Ti-6Al-4 V; femoral head in Co-Cr alloy (Stellite). The elements not highlighted come from the tissue and the embedding medium

ELEMENT	Mass Norm. ± 1 sigma [%]
Nitrogen	11.85 ± 0.76
Sodium	2.29 ± 0.09
Magnesium	1.45 ± 0.06
Aluminium	4.22 ± 0.11
Silicon	0.73 ± 0.04
Phosphorus	2.38 ± 0.06
Sulfur	2.22 ± 0.06
Chlorine	0.71 ± 0.04
Potassium	1.36 ± 0.04
Calcium	1.63 ± 0.05
Titanium	66.22 ± 0.75
Vanadium	1.21 ± 0.04
Iron	2.66 ± 0.06
Cobalt	0.14 ± 0.03
Copper	0.91 ± 0.04
SUM	100.00 ± 0.00

Once it has been verified that the elemental composition of the debris matches the sought material alloys, a map can be acquired from the same region, to more accurately locate the different fragments.

**Fig. 4:**
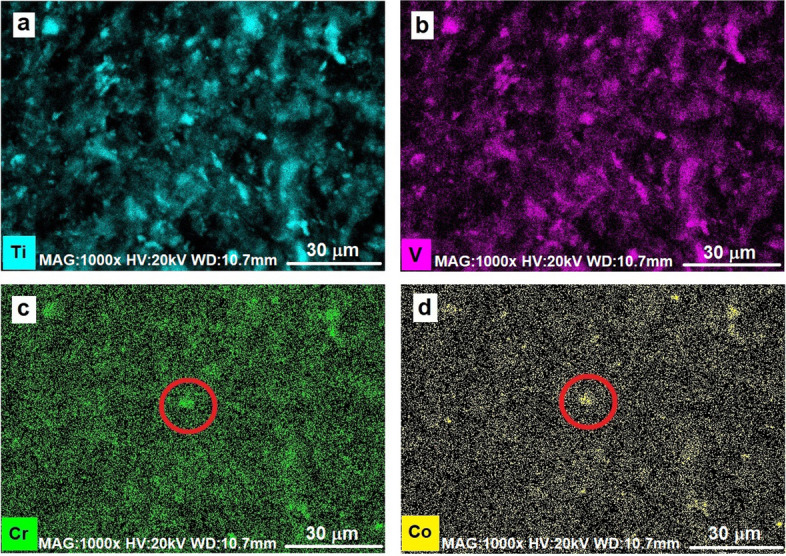
Full field EDX elemental mapping of Ti (**a**), V(**b**), Cr (**c**) and Co (**d**). In the maps for Cr and Co the circles indicate one of the several debris coming from a Stellite alloy

As the maps in Fig. 4a and b show, most of the debris are titanium alloy fragments from the acetabular cup. In some cases, they are mixed with Co-Cr based fragments, coming from the femoral head in Stellite alloy and observable where chromium and cobalt maps show higher concentrations (see circles in Figs. 4c and d).

**Fig. 5:**
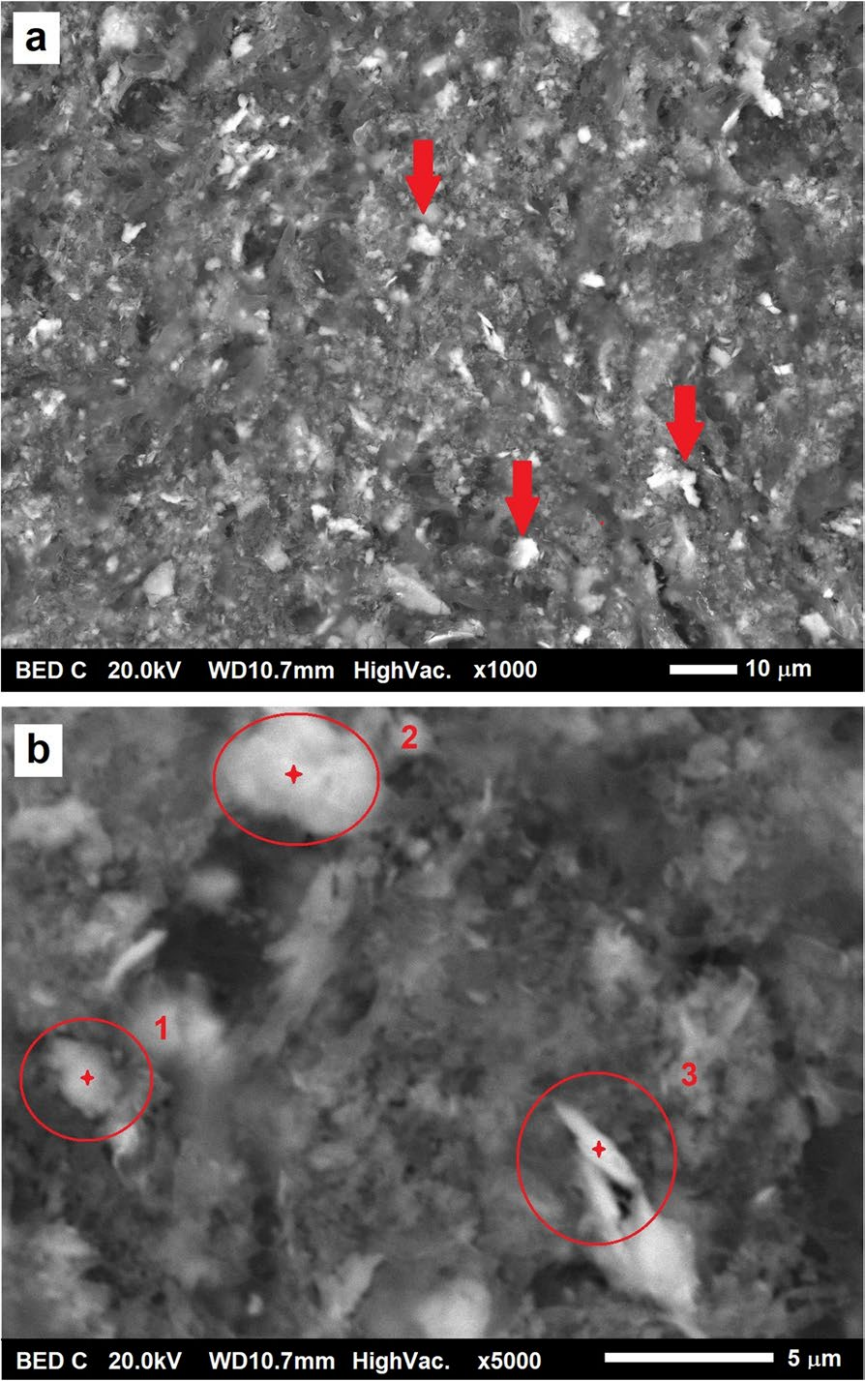
**a** Shows a BED image of the selected area of a KOH digested sample at medium magnification. The brighter particles, some of which indicated by red arrows, are metallic debris. **b** EDX acquisition spots marked in red. Spot 2 corresponds to the particle cluster providing a brighter contrast in the X-Ray Cr and Co maps (see the red circles of Fig. 4c and d)

For a better characterization of these regions, higher resolution EDX spectral analysis for spots is required (see Fig. 5b).

**Fig. 6:**
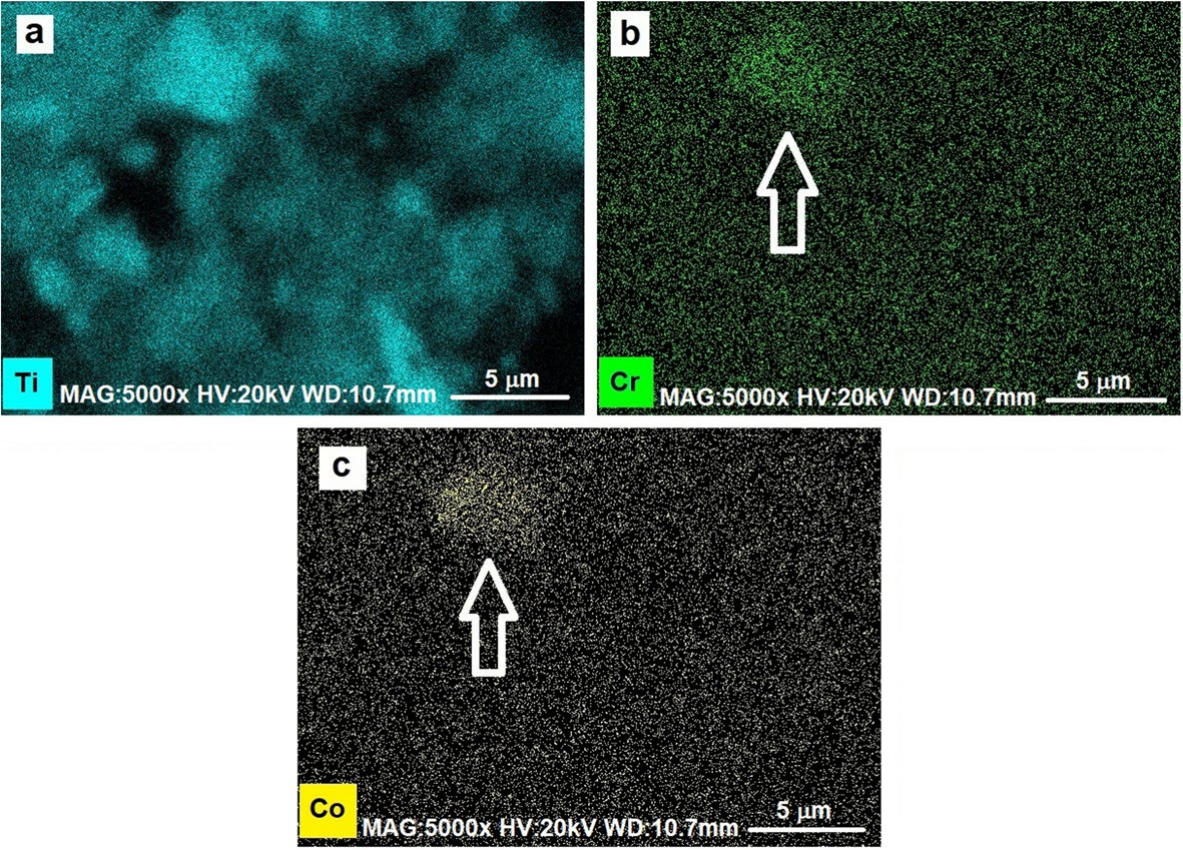
EDX elemental mapping of the zone containing the three spots. Only cluster in spot n.2 shows the presence of Cr-Co (pointed by the white arrow in **b** and **c**), the others appear as titanium alloy debris (**a**)

As shown by preliminary full-field EDX elemental mapping at higher magnification (Fig. 6), metal debris clusters in spots n.1 and n.3 consist mainly of titanium (in fact they are visible in Fig. 6a but absent in b and c). On the other hand, the cluster in spot n.2 shows the presence of Ti in association with Cr and Co too (see Fig. 6b and c, where cluster in spot n.2 only is in contrast—arrowed).

**Fig. 7:**
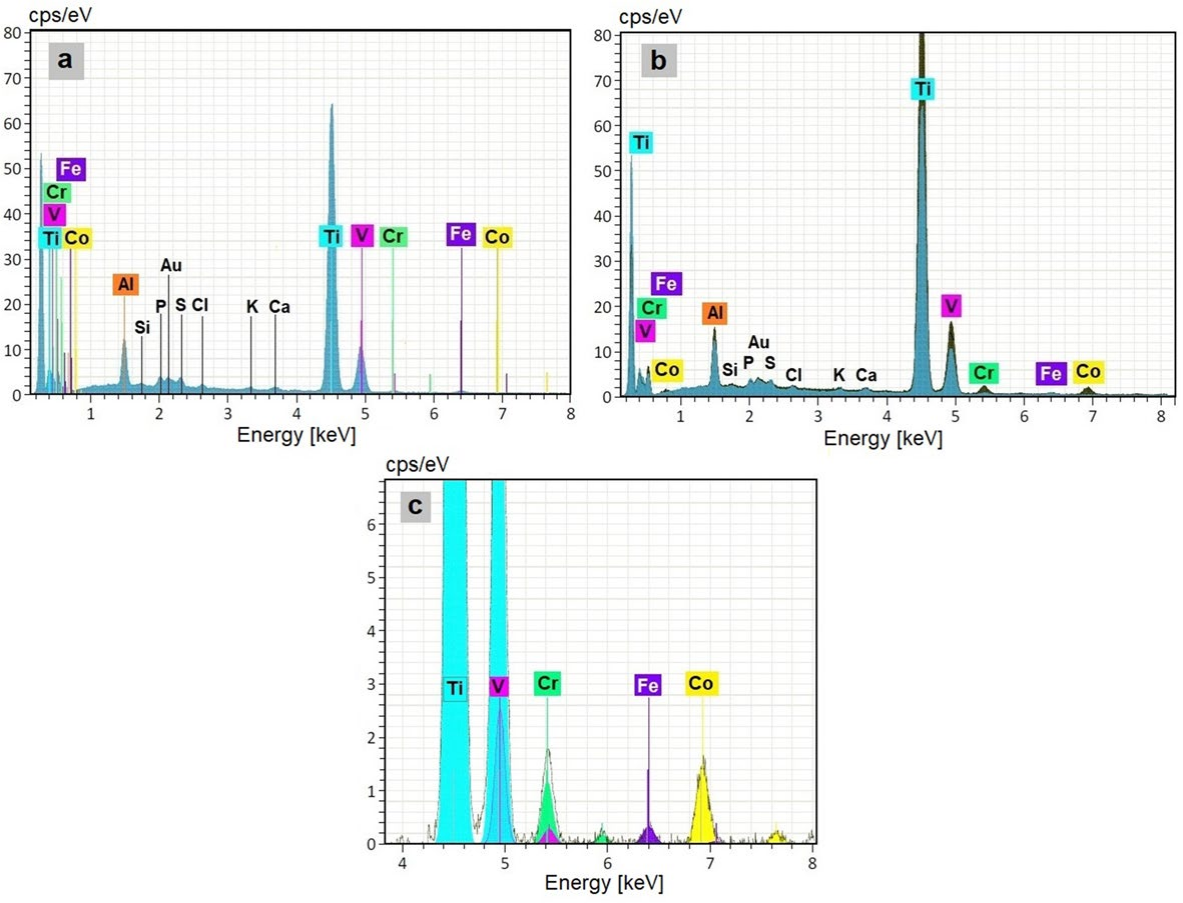
The spectral analysis results of spot n.1 reveal the absence of Cr and Co. Peaks related to Ti, Al, and V are evident. The less pronounced peaks related to Si, P, Au, S, Cl, K, and Ca are due to metallization (gold) and the presence of tissue and residues from the digestion process (the other elements). Spot n.3 shows similar spectrum (**a**). In (**b**), the spectrum of the cluster in spot n.1, in blue, is superimposed on the brown spectrum of the cluster in spot n.2. Notice in the brown spectrum the presence of the Cr and Co peaks, a detail of which and tabulated values are given below. **c** shows a detail, in the cluster n.2 spectrum, of the Cr (green) and Co (yellow) peak, next Ti, V and Fe peaks

EDX spectral analysis conducted on the individual spots confirms the absence of chromium and cobalt in spots n. 1 and n. 3, as shown in Fig. 7a, where the relevant peaks are those of titanium and vanadium.

By contrast, the EDX spectral analysis of the cluster in spot n. 2 contains both titanium alloy particulates and Cr-Co Stellites. In Table 2, Fig. 7b and c are the results and a detail of the peak related to Cr and Co.

**Table 2:** The tabulated results of the EDX spectral analysis of the cluster 2. It clearly shows the presence of Co and Cr

ELEMENT	Mass Norm. ± 1 sigma [%]
Aluminium	3.64 ± 0.18
Titanium	89.00 ± 2.12
Vanadium	2.26 ± 0.08
Chromium	1.47 ± 0.06
Cobalt	3.05 ± 0.10
Iron	0.58 ± 0.04
SUM	100.00 ± 0.00

When analyzing the spectra, it is often necessary to conduct a deconvolution of the signal. In our specific case the researched elements were Ti, Al and V, associated with the alloy constituting the acetabular cup and Cr and Co, related to the femoral head: therefore, special attention had to be paid to the peak overlapping of chromium and vanadium, since K_α_ (Cr) = 5.411 keV and K_β_ (V) = 5.426 keV (see Fig. 7c).

### 3rd SEM observation at high magnification and aspect ratio estimation

What at lower magnifications appear as single clear particles on a gray background are, at high magnification, clusters of particles, agglomerated in the periprosthetic tissue.

**Fig. 8:**
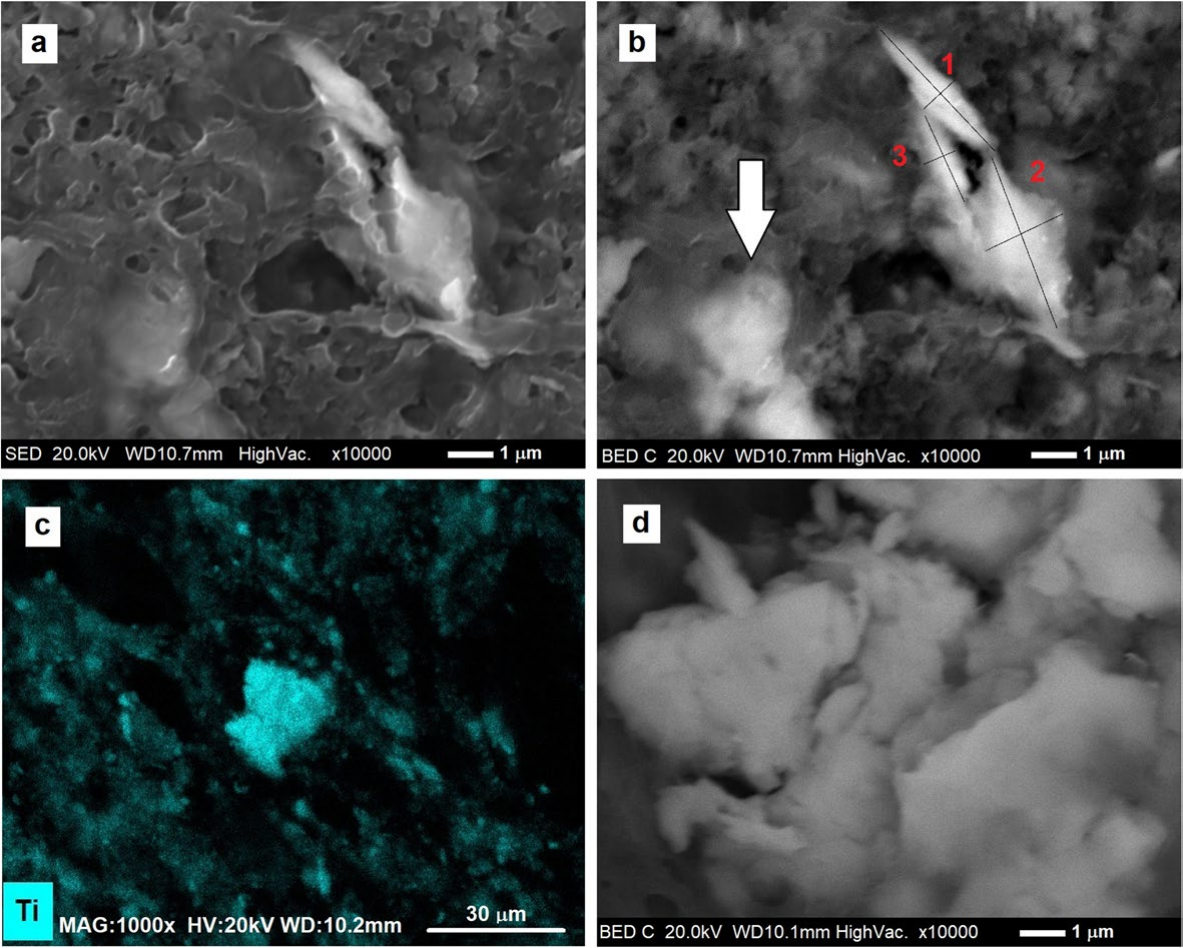
The SE picture (**a**) shows that the bottom left cluster is covered by the organic tissue, while the one on the right side of the image is emerging from the embedding tissue. From the BSE image (**b**), the major and minor axes can be evaluated to make an estimate of the particulate aspect ratio. The white arrow indicates a cluster excluded from the axis measurement. The full-field mapping of (**c**) shows the presence of a Ti alloy particle agglomerate. In (**d**) the high magnification image shows details of the Ti particles agglomerate of (**c**)

Comparing SE and BSE images some clusters appear particularly poorly emergent from the tissue, and it is therefore difficult to precisely distinguish the individual particles (see the one highlighted by the white arrow in Fig. 8a). For others, the aspect ratio of the constituent particles can be estimated, following axis measurements on BSE images.

The aspect ratio, defined as the ratio of the major axis to the minor axis, was calculated from 45 particles observed in tissue samples, digested with 0.2 M KOH solution. The axes were measured manually, as it was not possible, due to the presence of tissue, to take advantage of quantitative and morphological analysis programs such as ImageJ, since the particles are not isolated and distinct from each other. The effective length of the axes was proportional, for each image submitted for analysis, to the length of the reference bar, permitting a calculation of length in micrometers.

**Fig. 9:**
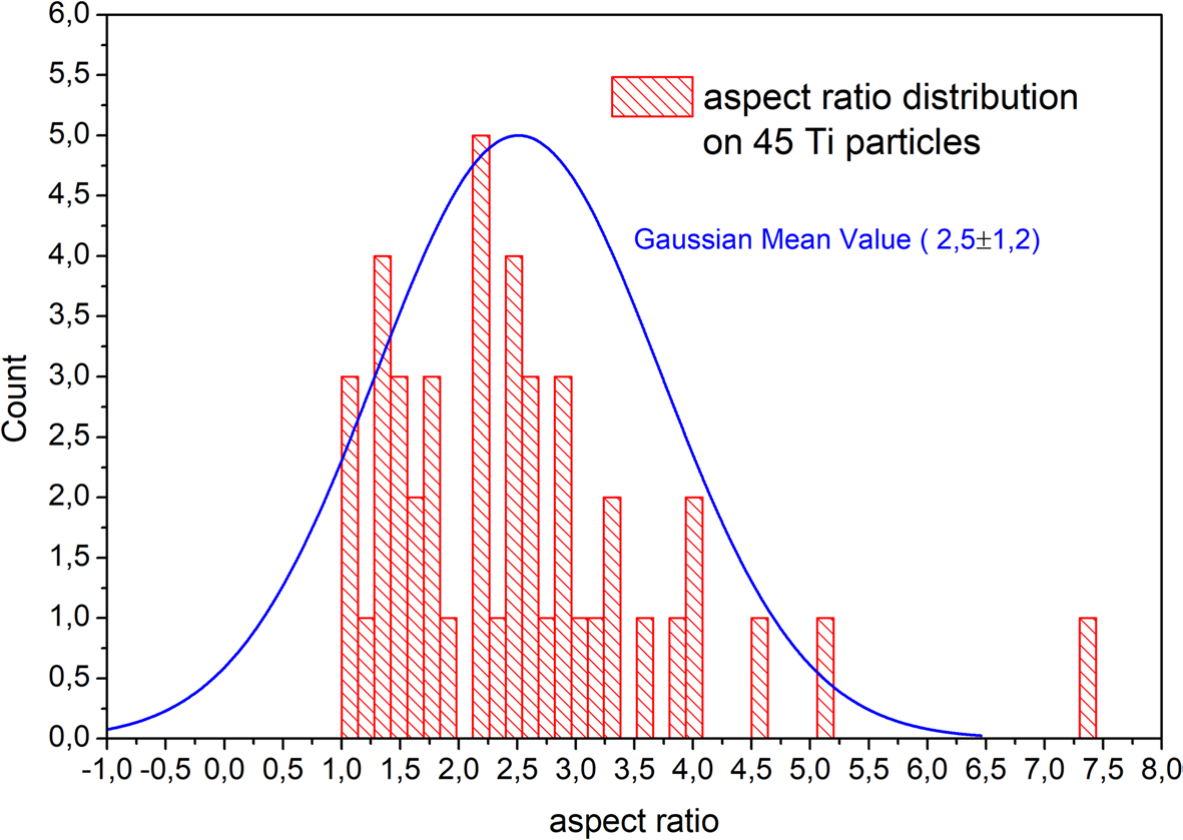
Gaussian distribution of the aspect ratio calculated on 45 Ti alloy particles

The value of the aspect ratio is (2.5 ± 1.2), associated to a range of [1.2: 7.3]. Figure 9 shows its distribution and Table 3 reports all the results obtained by the measurements carried out on the 45 particles.

**Table 3:** Tabulated results of measurements performed on 45 titanium particles. The red row is the one associated with the aspect ratio

TITANIUM PARTICLES (# 45 particles)	ASPECT RATIO	MAJOR AXIS [µm]	MINOR AXIS [µm]
MAX. VALUE	7.33	5.70	4.13
MIN. VALUE	1.10	0.90	0.33
RANGE	(from 1.10 to 7.33)	(from 0.90 to 5.70)	(from 0.33 to 4.13)
MEAN VALUE	2.5	2.2	1.0
ABS. ERROR (1 σ)	1.2	1.1	0.7

The aspect ratio greater than 1 defines the shape of the particles analyzed as rather elongated. No particles were found to have a spheroidal appearance, but neither were they elongated to the point of being defined as fibrillar. According to ASTM F1877 − 16 (2004), the shape of wear debris can be identified as granular, irregular, in some cases plate shape and shard or flake like. As visible from the SE and BSE images at high magnification, the surface is rather rough, it is however difficult to ascertain whether it is angular, either because of the presence of tissue or because the digestion process may have compromised the surface of the particulate matter.

Regarding the particle size, our results [2.5 ± 1.2 µm] are compatible with those found by Topolovec et al. [2.5 ± 3.6 µm in the first group of samples and 4.3 ± 2.8 µm in the second] in Topolovec et al. (2013). The higher magnifications we achieved, and the direct measurement of fragments observed individually allowed us to measure both isolated granules in the tissue and those distinguishable within clusters, avoiding two separate analyses. Figures 8b (see white arrow), 8.c and 8.d show some examples of clusters excluded from aspect ratio estimation.

In Topolovec et al. (2013) the clustering effect was observed almost exclusively for Cr-Co particles, not for Titanium particles. In our case, not only were numerous clusters found, but in most cases, they were clusters of Ti particles; only occasionally the presence of Cr-Co Stellite was also detectable [see Fig. 6 and related spectra].

This difference could be attributable to the fact that we conducted in situ analysis, but our sample, unlike those analyzed by Topolovec, underwent partial digestion, which may have partly changed the distribution of debris.

Another possible reason could be the particular reaction of the patient from whose tissue cells were able to conglomerate foreign bodies to respond to the inflammatory state.

## Discussion

The goal of the dimensional and morphological analysis of the observed debris is to understand the wear processes and mechanisms that led to particulate formation. The tribological study is important not only to understand the causes of prosthetic failure in the case under analysis, but also to help future research to develop more biocompatible implants with greater longevity, which is necessary given the increase in transplantation in the young and the increased life expectancy of the elderly.

Regarding the tribological origin of debris in artificial hip joints, in general the types of wear processes involved are mainly sliding wear and alternating sliding wear. The former is caused by the fact that the wear paths of the forward and reverse sections of the cycle do not lie on the same geometric lines, and the latter is due to the size of the contact area, which is smaller than the stroke of the wear path. As far as concern wear mechanisms, adhesion, abrasion, surface fatigue and tribochemical reactions are often present, even simultaneously (Merola and Affatato, 2019).

The biocompatibility of titanium and its alloys makes it the material of choice for the synthesis of prostheses. In fact, an artificial implant, once in the body, induces a cascade of effects in the surrounding biological microenvironment, due to the interaction of the biomaterial with body fluids, chloride ions, cells and proteins. Since implant failure is highly dependent on the nature of the interface that develops between the prosthesis and the surrounding tissue, most alloys used in prosthetic components tend to form a passive film that slows the corrosion process. Titanium alloys, in particular, form a very stable passive TiO_2_ layer that rebuilds quickly if damaged. This provides good resistance to pitting corrosion in vivo. However, other forms of corrosion are not similarly prevented by the TiO_2_ layer. These include fatigue corrosion, which is caused by low-frequency cyclic loading during walking or medium–high-frequency cyclic loading during running. There is also fretting corrosion, which is caused by relatively small reciprocating movements between the prosthesis components, which, although perfectly interlocked, are still in contact with body fluids due to these micro-movements.

Although the reputation of Ti and its alloys for corrosion resistance and biocompatibility is generally good, their performance for wear resistance is not, especially over long periods, and the safety of Al and V is of particular concern (Eisenbarth et al., 2004; Rao et al., 1996; Geetha et al., 2009; Watters et al., 2005; Budinski 1991; Eliaz 2018).

Long and Rack, in their study of the reciprocal sliding friction of various metastable beta alloys against hardened steel (Long et al., 2001), report SEM images of debris that are dimensionally and morphologically compatible with ours: the particles formed on the worn surfaces of the pins have an aspect ratio of less than 10 µm and a granular and irregular morphology. In the cases examined here, the alternative sliding motion induced microfracture mechanisms, surface plowing and localized asperity deformation, responsible for the formation of smaller debris. In contrast, larger debris formation was due to adhesive and transfer processes between titanium and steel.

Also Raj and Kailas in (2020), during dry sliding of Ti-6Al-4 V pins against SS316L under vacuum and ambient conditions, observed by SEM the presence of wear debris similar to those we detected. Specifically, these are plate-shaped, flake-like and fine/very fine wear debris (with widely varying sizes in the range of 2 to tens of µm) obtained under ambient conditions, principally by accumulation of plastic deformation and oxidative wear.

It should be noted that both articles report results from in vitro particulate samples obtained using specific tribometers and simulators. These can reproduce the main wear processes of a prosthesis but cannot replicate the effects of the biological environment surrounding it. In Long et al., (2001) and Raj et al., (2020) moreover, the wear of Ti alloys occurs against steel, whereas in our case against a Cr-Co head, misaligned with the worn Ti-6Al-4 V acetabular cup.

Factors such as the exact type of material involved, but mostly the environmental condition (vacuum, ambient or biological context of origin), the angle of inclination of the components and the type of movement between them create a major difference between the results obtained from in vivo and in vitro studies (Watters et al., 2005; Raj et al., 2020). For this reason, it is of particular interest to study in vivo the wear rate, morphology and tribological causes of debris produced by prosthetic implants.

We found agreement with the results obtained by Stratton Powell et al. in (2023), whose purpose was to determine the chemical composition, size, and morphology of wear debris surrounding failed total ankle replacements (TARs). Manual characterization of particulate matter detected by CFE-SEM and EDX, in 20 retrieved periprosthetic tissue samples from 15 failed TARs of different brands, revealed the presence of 487 Ti alloy particles with an aspect ratio of 2.25 ± 1.49 µm (our results: 2.5 ± 1.2 µm). According to this research, for the same material, particles generated by two different types of prostheses (Rebalance TAR and subluxed BP TAR) produced dimensionally different debris within the above range, due to different wear mechanisms. The micromotion at the implant-bone interface of the Rebalance TAR, wore the titanium alloy on the attachment surface and produced the largest debris. In contrast, metal-to-metal contact in the subluxed BP TAR, following displacement of the bearing insert, produced the smallest debris. These results were obtained after a process of complete digestion, the effects of which, combined with the inevitable corrosive action of the biological environment around the prosthesis, may have modified the morphology, which is nevertheless comparable to that observed in our study.

Considering the compatibility found with the aforementioned studies and the morphological and dimensional analyses conducted, we can hypothesize that the tribological origin of our debris is due to two main mechanisms. First, the sliding process between components promoted an adhesive mechanism between the softer titanium alloy and the harder chromium-cobalt alloy. This produced Stellite-containing debris, as seen in Fig. 4, where Ti, V, Cr, and Co maps match in some places, indicating the presence of all three elements within the same cluster. A second fretting wear process is associated with delamination of the titanium alloy and is responsible for generating particulate matter morphologically compatible with plates and flakes.

A more accurate tribological study can be carried out in the future, going to analyze, with this methodology, the wear to which the surfaces of prosthetic components have been subjected, comparing them with the type of wear obtained with appropriate tribological tests, tribometers and simulators (Fellah et al., 2014; Molinari et al., 1997; Chen et al., 2015; Topolovec et al., 2014; Kesteris et al., 2001).

## Conclusions

In this study we have set a detailed methodology for using effectively SEM and EDX analysis in the characterization of metal debris coming from the wearing out of periprosthetic implants. This methodology was applied to tissue samples taken after hip prosthesis explanations.

The novelty of the approach is the progressive stepwise increase of the resolution of the analyses, both imaging and spectroscopy tests, necessary for the identification of wear debris and of the relevant tribological phenomena intervening in their production.

The irregular, granular morphology and the micrometric dimensions of the observed fragments indicate that wear is determined in these mechanically reciprocating systems by the interplay of adhesion, abrasion and tribo-oxidation. Mechanisms such as contact fatigue and corrosion certainly also play an important role.

## Data Availability

The datasets used and/or analyzed during the current study are available from the corresponding author on reasonable request. Materials not available.
